# Optimizing the localization precision in coherent scattering microscopy using structured light

**DOI:** 10.1515/nanoph-2025-0435

**Published:** 2025-11-07

**Authors:** Ulrich Hohenester, Felix Hitzelhammer, Georg Krainer, Peter Banzer, Thomas Juffmann

**Affiliations:** Institute of Physics, 27267 University of Graz, Universitätsplatz 5, 8010 Graz, Austria; Biophysics, Institute of Molecular Biosciences (IMB), University of Graz, NAWI Graz, Graz, Austria; Field of Excellence BioHealth, University of Graz, Graz, Austria; Faculty of Physics, VCQ, University of Vienna, 1090 Vienna, Austria; Max Perutz Labs, University of Vienna, 1030 Vienna, Austria; Christian Doppler Laboratory for Structured Matter Based Sensing, Institute of Physics, Graz, Austria

**Keywords:** optical coherence microscopy; Fisher information; structured light

## Abstract

We employ the concept of quantum Fisher information to optimize the focused excitation fields in coherent scattering microscopy. Our optimization goal is to achieve the best possible localization precision for small scatterers located above a glass coverslip, while keeping the intensity of the total incoming excitation fields fixed. For small numerical aperture (NA) values, the optimal fields have linear or circular polarization, and the excitation beam can be well approximated by a Gaussian one. For larger NA values, the optimal beam acquires radial polarization. We show that the high localization precision can be attributed to high field strengths at the scatterer position, and correspondingly a large number of scattered and detected photons. Finally, we evaluate the performance of the optimized beams in interferometric scattering microscopy (iscat), and further optimize these fields for iscat localization using the concept of Fisher information.

## 1 Introduction

Coherent optical microscopy techniques, such as optical coherence tomography, holography, and interferometric scattering (iscat), have a profound impact on science and technology [1], [2]. The precision with which a scattering particle can be localized and tracked is often an essential figure of merit in these techniques.

In many modern microscopes, the noise in an image is dominated by shot-noise, and the localization precision scales as $1/\sqrt{N}$ , where *N* is the number of detected photons [3]. While increasing *N* leads to higher precision, this is often not possible, mostly due to either detector limitations (finite frame rate and well depth) [4] or the sample being negatively affected by a high photon fluence. Note that the latter has to be considered both in fluorescence and also in label-free microscopy [5], [6].

In such cases, it is crucial to maximize the information obtained per photon. This can be assessed using the concepts of (quantum) Fisher information and (quantum) Cramer–Rao bounds [3], [7]. Substantial work has been devoted to the localization precision for incoherent light sources [8], [9], [10], [11]. Regarding coherent optical microscopy techniques, Bouchet et al. [12] quantitatively compared the phase estimation precision of various phase microscopy techniques, while related work quantified the precision achievable in estimating the position and polarizability of scattering particles [13] and extended these studies to the optical near-field [14]. Experimentally, the framework was applied to optimize axial localization in iscat [15] and off-axis iscat in the situation when defocus is not desired [16]. Similar studies were done in electron microscopy [17], [18], [19], where dose-induced specimen damage limits the spatial resolution in biological imaging.

Importantly, these studies assumed either plane-wave or scanned-focus illumination, trying to find an optimal detection modality such that the Fisher information comes as close as possible to the fundamental limit of the quantum Fisher information. This approach neglects the possibility of shaping the illumination light in order to maximize the quantum Fisher information.

The benefits of using a shaped illumination has been explored in fluorescence microscopy, in particular in the context of minflux, where it has been found that the (quantum) Fisher information per fluorescence photon can be vastly increased with an illumination featuring zero intensity at the position of the fluorophore [20], [21], [22], [23]. Positioning the illumination zero at various positions close to the fluorophore enables the precise localization based on a few detected fluorescence photons. Importantly, the information is quantified per fluorescence photon, as it is the number of emitted photons that limits estimation precision, determines phototoxicity, and limits observation time due to bleaching.

Recently, shaped illumination has also been used to identify optimal coherent measurements on scatterers inside highly scattering media [24], [25]. This work also led to optimal states for the optical manipulation of particles [26] and to a general framework of information flow [27].

Here, we study the optimization of localization precision in coherent microscopy techniques based on shaping the incoming illumination. In contrast to the existing work on fluorescence microscopy, we optimize the (quantum) Fisher information per incoming photon and not per scattered photon. This is justified in applications where phototoxicity, e.g., via the excitation of endogenous fluorophores [6], limits the illumination intensity. This regime gains more and more importance as the spatial and temporal resolution of coherent microscopy techniques is improved, often at the price of higher illumination intensities.

We first compare the two most common iscat modalities, which feature either focused excitation scanned across the sample or widefield excitation. We then optimize the illumination using a fast and powerful matrix optimization approach, which yields optimal solutions that maximize the quantum Fisher information in the collected light, or the Fisher information in a specific measurement. While the optimal solutions depend on the numerical aperture (NA) of the detection system, we find that a simple focused Gaussian beam performs close to optimally, particularly for smaller NAs. At high NAs, we find that optimized structured illumination provides a more than twofold improvement in quantum Fisher information. At very high NAs, and for particles located close to an interface (as is the case in many iscat measurements), we further find that evanescent modes also contribute significantly to the localization precision.

## 2 Theory

**Figure 1:**
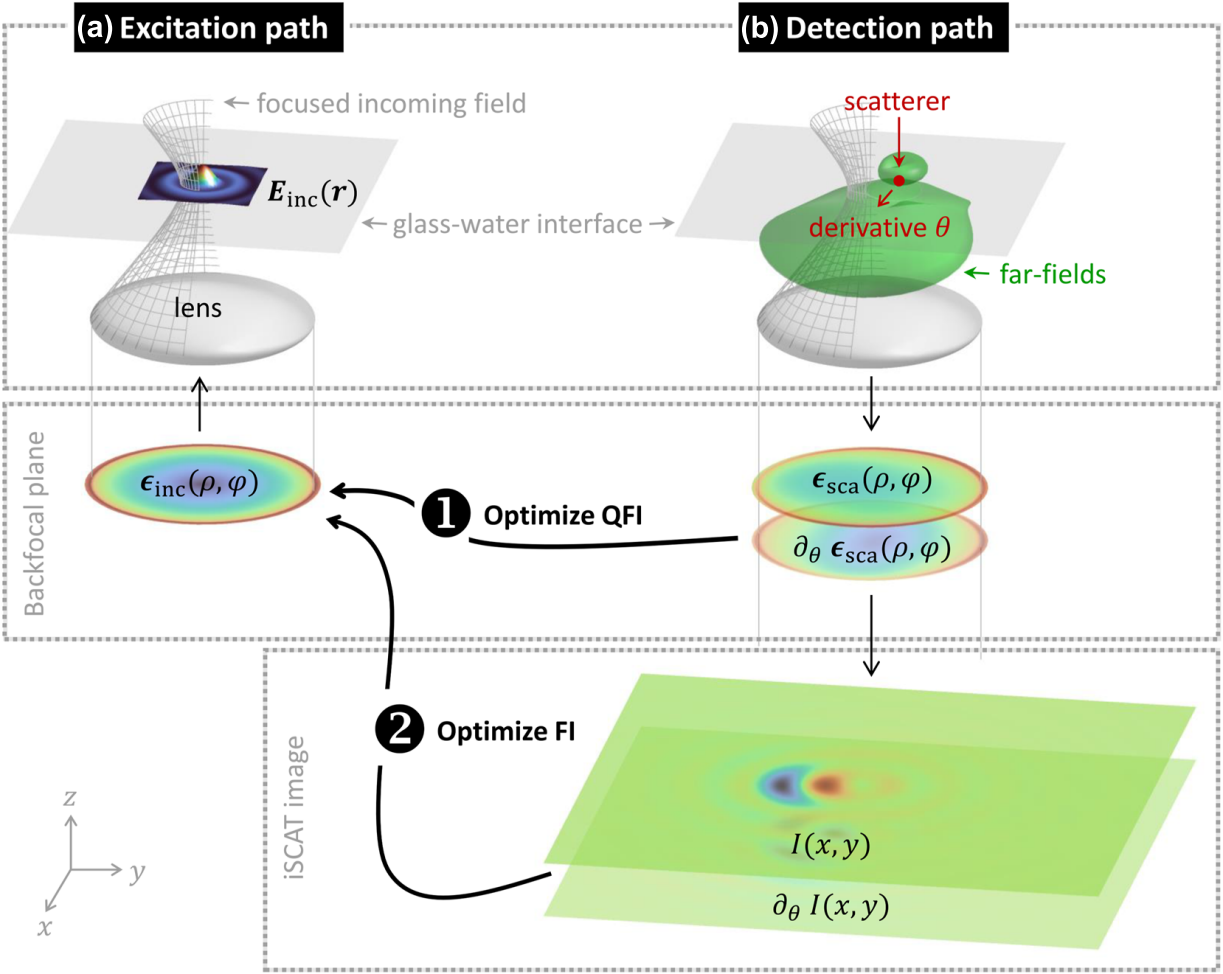
Schematics of optimization of Fisher information (FI) and quantum Fisher information (QFI) in coherence scattering microscopies. The objective of the optimization is to obtain the best localization in (1) any type of coherence microscopy and (2) interferometric scattering microscopy (iscat). (a) An incoming field ***ϵ***_inc_ propagating along +*z* in the backfocal plane is focused onto a glass–water interface. (b) A particle located on top or above the interface scatters light (see far-field pattern), which is collected by the same lens and transformed to a backfocal scattered field ***ϵ***_sca_ propagating along −*z*. Together with the incoming fields reflected at the glass–water interface (reference fields), the superimposed fields form the iscat image I(x,y) . (1) The variation of the scattered backfocal fields ∂_*θ*_ ***ϵ***_sca_ with respect to an estimation parameter *θ*, here the particle position along *x*, allows to compute the quantum Fisher information and to optimize the incoming fields ***ϵ***_inc_. (2) Similarly, the variation of the iscat image $\partial_{\theta }I\left(x,y\right)$ allows to compute the Fisher information and to obtain the optimized incoming fields for particle localization based on iscat. For details, see text.

The problem of our present concern is depicted in Figure 1. An incoming light beam propagating in the backfocal plane along +*z* is focused onto a particle (denoted as the scatterer) located at position ***R***_0_,

(1) $$\epsilon_{\text{inc}}\left(\rho ,\varphi \right)\to E_{\text{inc}}\left(R_{0}\right).$$

We will consider the situation that the scatterer is embedded in water and is either located directly on top of a glass coverslip or sufficiently far away from it. Part of the incoming light is coherently scattered by the particle, the far-fields ***F***_sca_ propagating in the direction $\hat{r}$ toward the focusing lens are subsequently collected and are accessed in experiment either in the backfocal plane or as imaged fields [28],

(2) $$\frac{{\text{e}}^{\text{i}\mathrm{kr}}}{r}F_{\text{sca}}\left(\hat{r};R_{0}\right)\to \epsilon_{\text{sca}}\left(\rho ,\varphi ;R_{0}\right)\to E_{\text{sca}}^{\text{im}}\left(x,y;R_{0}\right).$$

Together with the reference fields ***E***_ref_, which are reflected at the glass–water interface of the setup, one can compute the iscat image from

(3) $$I\left(x,y;R_{0}\right)={\left|E_{\text{sca}}^{\text{im}}\left(x,y;R_{0}\right)+E_{\text{ref}}^{\text{im}}\left(x,y\right)\right|}^{2}.$$

The questions we will address in this work are as follows:

1. What are the optimal incoming fields $\epsilon_{\text{inc}}^{\left(opt\right)}$ for obtaining the best possible localization precision of a scatterer using an optimal localization protocol?
2. What is the best possible localization precision that can be obtained on the basis of the iscat images?

### 2.1 Fisher information

To answer these questions, we will use the concept of the Fisher information (FI) [3], [7]. It accounts for the amount of information that the measurement data carry about an unknown parameter. Let us first consider the localization estimation using experimental iscat data. Our goal is to estimate the particle position ***R***_0_ from the iscat images $I\left(x,y;R_{0}\right)=I\left(x,y;\theta \right)$ that are noisy (Poisson noise) because of the limited number of photons collected [7]. We have followed the usual convention adopted in Fisher information theory and have denoted the estimation parameters with ***θ***. In the following, we will only consider localization estimation, this is ***θ*** = ***R***_0_, but our approach could be easily modified to deal with different estimation parameters as needed, e.g., in mass photometry or the discrimination of closely spaced scatterers [29].

Suppose that we have two different incoming fields ***ϵ***_inc_ at hand. Through the Fisher information, we can determine which field provides greater sensitivity to the parameter of interest and should thus be used in noisy experiments. In the language of probability theory, $p_{I}\left(x,y;\theta \right)$ is a (Poisson) *probability *mass* function whose expectation value gives* $I\left(x,,,y,;,\theta \right)$ . We next introduce the *score*

(4) $$\text{Score}\left(\theta_{i}\right)=\frac{\partial_{\theta_{i}}p_{I}\left(x,y;\theta \right)}{p_{I}\left(x,y;\theta \right)},$$

which accounts for the (normalized) change of $p_{I}$ when varying the estimation parameter *θ*_*i*_, see also Figure 1. The Fisher information is defined as the variance of the score,

(5) $$FI\left(\theta_{i}\right)=E\left({Score}^{2}\left(\theta \right)\right)=E\left({\left[\frac{\partial_{\theta_{i}}p_{I}\left(x,y;\theta \right)}{p_{I}\left(x,y;\theta \right)}\right]}^{2}\right).$$

A large Fisher information indicates that the measurement data change significantly upon variation of *θ*_*i*_, and thus a high localization precision can be obtained in experiment. The expectation value E in Eq. (5) has the form [7], [13]

(6) $$FI\left(\theta_{i}\right)=\frac{1}{N}\left(\underset{x,y}{\sum }\frac{{\left[\partial_{\theta_{i}}I\left(x,y;\theta \right)\right]}^{2}}{I\left(x,y;\theta \right)}\right)\Delta x\Delta y,$$

where the sum extends over the pixels *x*, *y* of the image detector and Δ*x*, Δ*y* denote the pixel size. We have introduced a normalization factor *N* that is conveniently set to the intensity of the scattered fields in the backfocal plane [13] (proportional to the number of detected photons). In the study of structured light fields, we proceed somewhat differently and use instead the intensity of the incoming fields (proportional to the number of incoming photons). From the Fisher information, one can define the Cramér–Rao bound on the standard deviation of any unbiased estimator for the parameter *θ*_*i*_ per incoming photon as

(7) $$\sigma_{\text{inc}}^{\text{iSCAT}}\left(\theta_{i}\right)=\frac{1}{\sqrt{FI\left(\theta_{i}\right)}}.$$

The equality sign should be understood as an assignment of notation, where $\sigma_{\text{inc}}^{\text{iSCAT}}$ sets a lower bound for the standard deviation achievable from any estimator saturating the Cramér–Rao bound. The corresponding bound per detected scattered photon is related through

(8) $$\sigma_{\det}^{\text{iSCAT}}\left(\theta_{i}\right)=\sqrt{\frac{N_{\det}}{N_{\text{inc}}}}\sigma_{\text{inc}}^{\text{iSCAT}},$$

where *N*_inc_ and *N*_det_ denote the numbers of incoming and detected photons, respectively.

### 2.2 Quantum Fisher information

We can extend the concept of Fisher information to the quantum regime, where the quantum Fisher information (QFI) quantifies the sensitivity of a quantum state to changes in the parameter of interest, independent of any specific measurement scheme. In other words, it doesn’t rely on iscat or any other variant of coherence microscopy but addresses the question what could be obtained under the best possible measurement conditions. As has been shown in Ref. [24], for coherent scattering measurements (as studied in this work), the quantum Fisher information can be obtained from the scattered fields in the backfocal plane through

(9) $$QFI\left(\theta_{i}\right)=\frac{4}{N}\overset{\rho_{\text{NA}}}{\underset{0}{\int }}\overset{2\pi }{\underset{0}{\int }}{\left|\partial_{\theta_{i}}\epsilon_{\text{sca}}\left(\rho ,\varphi ;\theta \right)\right|}^{2}\;d\varphi \rho d\rho ,$$

where *ρ*_NA_ is the cutoff radius for a given numerical aperture (NA) of the lens. Below we will show that these optimized fields can be obtained in a computational approach through a matrix diagonalization, which makes the approach extremely simple and powerful. In the same way as for the Fisher information discussed above, we can introduce a quantum Cramér–Rao bound

(10) $$\sigma_{\text{inc}}^{\text{quant}}\left(\theta_{i}\right)=\frac{1}{\sqrt{QFI\left(\theta_{i}\right)}},$$

which sets a lower bound on the standard deviation of the best possible estimator for the parameter *θ*_*i*_ and per incoming photon.

### 2.3 Normalization of Fisher information

When normalizing the Fisher information, we can do so in many ways. We can normalize per incoming photon, per scattered photon, per absorbed photon, or per detected photon. In order to choose the right normalization, we have to know what limits the accuracy in our measurements experimentally. Here are some examples:

1. If the number of photons per time available from the light source is limiting the experiments, then one should optimize per incoming photon. This is rarely a problem these days.
2. In fluorescence microscopy, it is known that the excitation, relaxation, and photobleaching of the fluorophores lead to reactive oxidative species that can harm live cells and organisms [30]. It is, therefore, often appropriate to maximize the Fisher information per excitation–relaxation cycle, i.e., per emitted fluorescence photon.
3. In coherent microscopy, phototoxicity is often less of a problem, and many experiments are limited by the low photon detection rate, i.e., the number of photons that can be recorded per pixel per frame. In this case, one should normalize per detected photon, which we did in a previous paper [13].
4. However, even if an imaging modality is based on elastic scattering, the illumination light can still cause phototoxicity, e.g., due to molecules that absorb light. A common example is the imaging of live-cells with blue or UV light, but even infrared light can inactivate bacteria at moderate intensities [31], which are commonly applied in coherent scattering microscopy. In this case, it is then again appropriate to normalize per incoming photon. With coherent scattering imaging being done at faster and faster framerates, requiring higher and higher excitation intensities [4], this regime will become more prominent in the future, which is why we chose this normalization for our manuscript.

### 2.4 Computational approach

In our computational approach, we adopt the coherent scattering microscopy add-on [28] to the generic Maxwell solver toolbox nanobem [32], [33]. Contrary to our previous work, we are only interested in small and weak scatterers that can be described well in the dipole approximation [34] without solving the full Maxwell equations for the dielectric particle. All our simulations can be broken down into the following steps (see [28] for a more detailed discussion).

1. We start by specifying the incoming fields on a polar grid, ***ϵ***_inc_(*ρ*_*m*_, *φ*_*m*_), which become focused toward the interface using the Richards–Wolf approach [35], [36] in order to obtain the fields ***E***_inc_(***R***_0_) at the scatterer position ***R***_*o*_.
2. The incoming fields induce an oscillating dipole moment ***p*** = *α* ***E*** of the scatterer, where *α* is the polarizability of a sphere [34], which produces outgoing waves $\frac{{\text{e}}^{\text{i}\mathrm{kr}}}{r}F_{\text{sca}}\left(\hat{r}\right)$ in the optical far-field [28], Eq. (6)] (see also far-field pattern in Figure 1). Note that in coherent scattering microscopy and for spherical scatterers, the dipole moment always points in the same direction as the excitation field.
3. The scattered fields are collected by a lens and converted to fields ***ϵ***_sca_(*ρ*_*m*_, *φ*_*m*_) propagating in the backfocal plane along −*z*. Again, the fields are given on a polar grid.
4. Using the Richards–Wolf approach for imaging, we compute the scattered and reference fields in the image plane [28]. The intensity of the superposition of these fields then provides us with the iscat image $I\left(x,y;R_{0}\right)$ , see Eq. (3).

We are now ready to compute both types of Fisher information. We first approximate the derivative of the scattered fields in the backfocal plane using finite differences.

(11) $$\partial_{\theta_{i}}\;\epsilon_{\text{sca}}\left(\theta \right)\approx \frac{1}{\eta }\left[\epsilon_{\text{sca}}\left(\theta +\eta \;{\hat{e}}_{\theta_{i}}\right)-\epsilon_{\text{sca}}\left(\theta \right)\right],$$

with a similar expression for $\partial_{\theta_{i}}I\left(x,y;\theta \right)$ . Here *η* is a small number and ${\hat{e}}_{\theta_{i}}$ is the unit vector along the direction *θ*_*i*_. This is depicted in Figure 1(b) for *θ*_*i*_ = *X*_0_, where the scatterer is slightly displaced along *x* and the scattered far-fields are computed for the displaced scatterer. For the integral over the backfocal plane, see Eq. (9), we use a polar grid to get for a generic function F(ρ,φ) the expression

$$\overset{\rho_{\text{NA}}}{\underset{0}{\int }}\overset{2\pi }{\underset{0}{\int }}F\left(\rho ,\varphi \right)\;d\varphi \rho d\rho \approx \underset{m}{\sum }w_{m}F\left(\rho_{m},\varphi_{m}\right),$$

where *w*_*m*_ are the quadrature weights adopted in the numerical approach, such as Gauss–Legendre weights in this work. We next use that in linear response the scattered fields can be related to the incoming fields through a transition matrix ***T*** (***θ***),

(12) $$\epsilon_{\text{sca},m}=\underset{m^{\prime }}{\sum }T_{mm^{\prime }}\left(\theta \right)\cdot \epsilon_{\text{inc},m^{\prime }},$$

where *m* labels the polar grid points and we have introduced the short-hand notation ***ϵ***_*m*_ = ***ϵ*** (*ρ*_*m*_, *φ*_*m*_). In computing the quantum Fisher information, we set the normalization factor to the intensity of the incoming fields, *N* = ∑_*m*_ *w*_*m*_| ***ϵ***_inc,*m*_|^2^, and obtain from Eq. (9)

(13) $$\epsilon_{\text{inc}}^{†}\cdot \left[4\left(\partial_{\theta_{i}}T^{†}\right)\cdot \left(\partial_{\theta_{i}}T\right)\right]\cdot \epsilon_{\text{inc}}=QFI\left(\theta_{i}\right)\;\left(\epsilon_{\text{inc}}^{†}\cdot w\cdot \epsilon_{\text{inc}}\right),$$

with the diagonal matrix ***w***_*mm*′_ = *w*_*m*_ *δ*_*mm*′_. Equation (13) is a quadratic form, and the best possible fields are obtained from the solutions of the generalized eigenvalue problem (see [24] for a related approach)

(14a) $$F_{\theta_{i}}\cdot \epsilon_{\text{inc}}=\Lambda \;w\cdot \epsilon_{\text{inc}},$$

(14b) $$F_{\theta_{i}}=4\left(\partial_{\theta_{i}}T^{†}\right)\cdot \left(\partial_{\theta_{i}}T\right).$$

The eigenvectors associated with the largest eigenvalue Λ = QFI(*θ*_*i*_) are the optimized fields $\epsilon_{\text{inc}}^{\left(opt\right)}$ with the best possible localization precision. Instead of optimizing for a single localization parameter *θ*_*i*_, in this work we replace the matrix $F_{\theta_{i}}$ with

(15) $$F{=F}_{X_{0}}{+F}_{Y_{0}}+{\gamma \;F}_{Z_{0}},$$

where ***R***_0_ = (*X*_0_, *Y*_0_, *Z*_0_) is the particle position. Using this expression, in the diagonalization, we then optimize the localization precision for all three spatial directions rather than a single one, where *γ* is a scaling parameter that weights the relative importance for localization along *X*_0_, *Y*_0_ with respect to *Z*_0_. Quite generally, one could also consider off-diagonal contributions to the Fisher matrix, but for the setups considered in this work, they turned out to be of minor importance and were thus neglected.

For iscat experiments, the optimization of the FI(*θ*_*i*_) in Eq. (5) cannot be expressed as a quadratic form in the incoming fields and cannot be converted to an eigenvalue problem or something related. We thus have to proceed differently. In our computational approach, we start from some initial guess and maximize the Fisher information in an iterative fashion using the fminunc function of matlab. More details about our approach will be given in Section 3.3.

## 3 Results

Using the formalism presented in Section 2, we performed optimizations of the Fisher information for a weak scatterer located on top of a glass–water interface or placed sufficiently far away from it. The pertinent simulation parameters are listed in Table 1. We consider a weak scatterer, which we model as a sphere with a diameter of 20 nm and a refractive index of 1.58, representative for biological samples [37]. In our simulations, we employ the dipole approximation for the light scattering of the particle, which is then fully characterized by its polarizability. For this reason, the precise particle parameters are of no particular importance in our simulation results, apart from a constant prefactor for the total scattered fields that are proportional to the polarizability of the scatterer. We correspondingly report the Fisher information and all related quantities in arbitrary units throughout. Our results would not change dramatically when the particle shape slightly deviates from a spherical shape, at least for aspect ratios close to one. For the small permittivity contrasts considered in this work, multiple scatterings between the particle and the substrate are expected to be of minor importance, although they could be added in our approach along the lines discussed in Ref. [28]. Note that for the scatterer on top of the glass–water interface, we use an additional gap distance of 5 nm, but the results would remain practically unaltered when locating the particle directly on the interface. In all our simulations, we use our previously published software nanobem [28], [38].

**Table 1:** Simulation parameters used in this work.

Symbol	Value	Description
*λ*	520 nm	Light wavelength in vacuum
NA	1.0–1.45	Numerical aperture
*n*_*ρ*_, *n*_*ϕ*_	25–100	Discretization points for lenses
*n*_glass_	1.50	Refractive index for glass
*n*_water_	1.33	Refractive index for water
*n*_part_	1.58	Refractive index for particle
*d*	20 nm	Diameter of particle
*z*_0_	5 nm, 1 μm	Particle–interface distance

### 3.1 A first glimpse on optimization

**Figure 2:**
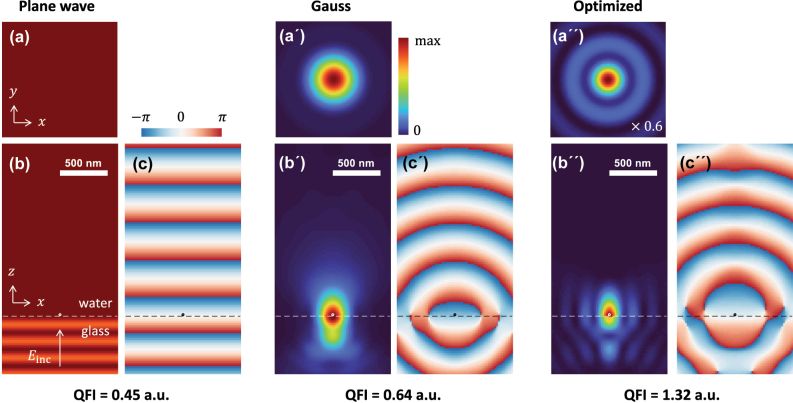
Intensity and phase maps for coherent scattering microscopy using a plane wave (left), a focused Gaussian beam (middle), and an optimized beam (right). The focusing lens has a numerical aperture of NA = 1.4. We report the intensity of the incoming beam in the (a) *xy* (*z* = 0) and (b) *xz*-plane. The glass–water interface is indicated by a dashed line, and we also show the scatterer with a diameter of 20 nm located on top of the interface. Panel (c) reports the phase map for the incoming field *E*_inc,*x*_. Below the figures, we report the quantum Fisher information in arbitrary units. In the simulations, we use the same incoming intensities for the Gauss and optimized beam, the field strength of the plane wave is chosen such that it equals that of the Gauss beam at the position of the scatterer. The intensity of the optimized beam is scaled by a factor of 0.6.

In order to get a better feeling of what we are aiming for, in Figure 2, we plot the field intensities and phase distributions for three incoming fields, namely a plane wave impinging from below on the glass–water interface (left, a–c), a focused Gauss beam (middle, a’–c’), and an optimized beam (right, a”–c”) that is obtained from the maximization of the quantum Fisher information in Eq. (14). For the plane wave polarized along *x*, we observe below the interface the interference between the primary incoming wave propagating in the +*z* direction and the secondary wave reflected at the interface propagating in the −*z* direction. In the middle panels, we show the results for a Gaussian incoming field with circular polarization

(16) $$\epsilon_{\text{inc}}\left(\rho \right)=E_{0}\exp\left(-\frac{\rho^{2}}{2}\right)\frac{\hat{x}+i\hat{y}}{\sqrt{2}},$$

which becomes focused through a lens with a numerical aperture of NA = 1.4 (see Figure 1). *E*_0_ is chosen such that the field strengths of the plane wave and the focused Gauss beam are the same at the scatterer position ***R***_0_. When comparing the quantum Fisher information values of Eq. (9), reported at the bottom of Figure 2, we observe that the focused Gauss beam has a better localization precision. In the right panels, we show that the localization precision can be further boosted through an optimization of the quantum Fisher information. This shows that, with constant electric field at the scatterer position, a narrow illumination beam yields the highest quantum Fisher information regarding localization in the scattered light. This result has to be met with caution. First, with a tightly focused illumination beam, it will be difficult to tune the scattered and the reference fields separately (e.g., via defocusing for phase, or via the use of attenuators for amplitude tuning), potentially leading to the Fisher information being significantly smaller than the quantum Fisher information. Second, if a focused beam is scanned in order to image an extended field of view, the average quantum Fisher information will probably be lower for the focused beams.

### 3.2 Optimization of quantum Fisher information

**Figure 3:**
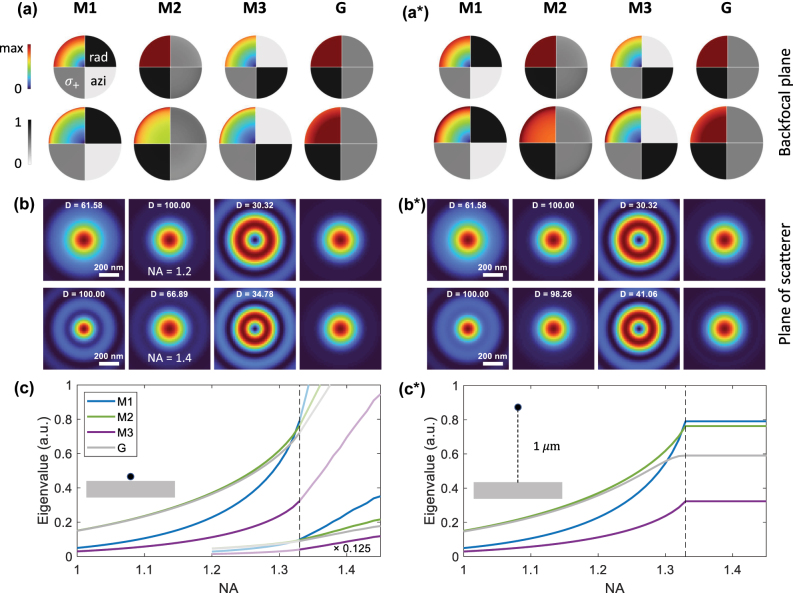
Modes with optimized quantum Fisher information in (a, a*) backfocal plane and (b, b*) plane where the scatterer is located, and for scatterer (a, b) on top of glass–water interface and (a*, b*) located 1 μm away from the interface. The colored quarter-disks in (a, a*) show the intensities of the incoming beams, and in the grayscale quarter-disks, we project the field-distribution onto radial, azimuthal, and circularly polarized (*σ*_+_) unit vectors. The optimized modes exhibit (M1) radial, (M2) circular, and (M3) azimuthal polarization. For comparison, we also show a (G) Gaussian mode with circular polarization. The modes in the first and second rows of panels (a, b) show results for numerical apertures of 1.2 and 1.4, respectively. (c, c*) Eigenvalues of Eq. (14), which are proportional to the quantum Fisher information, as a function of NA for different modes. For the particle on top of the substrate and for large NA values, we scale the eigenvalues by a factor of 0.125 for better visibility.

In this section, we provide a detailed discussion of the optimized structured-light beams and the resulting quantum Fisher information values. We start from Eqs. (14a) and (15) with *γ* = 0.2 and comment on the importance of the scaling parameter *γ* in Appendix A. Figure 3 shows the optimized incoming modes in (a, a*) the backfocal plane and (b, b*) the plane where the scatterer is located. The left panels (a–c) report results for the scatterer on top of the interface, and the right ones (a*–c*) for a particle–interface distance of 1 μm. In the first rows of panels (a, a*) and (b, b*), we show results for a numerical aperture of NA = 1.2, and in the second rows for NA = 1.4.

Although in our optimization approach we do not enforce any symmetry constraints, the problem under study exhibits cylinder symmetry, mainly due to our consideration of small particles (note that things would strongly differ for larger particles [39]), and thus also the optimized solutions exhibit such symmetry. For this reason, we plot in Figure 3(a) the field intensities and polarization properties only in a quarter of a disk. The colored parts (upper-left quarter-disks) report the intensities of the optimized incoming fields ***ϵ***_inc_(*ρ*, *ϕ*) in the backfocal plane. In the remaining quarter-disks, we project the fields onto unit polarization vectors ***u***_*λ*_ through

(17) $$P\left(\rho ,\phi \right)=\frac{\left|u_{\lambda }^{*}\left(\rho ,\phi \right)\cdot \epsilon_{\text{inc}}\left(\rho ,\phi \right)\right|}{\left|\epsilon_{\text{inc}}\left(\rho ,\phi \right)\right|},$$

where the degree of polarization P is bound to values between zero and one. In the figure, we use (in clockwise direction) polarization vectors in the radial and azimuthal directions, as well $u_{\pm }=\left(\hat{x}\pm i\hat{y}\right)/\sqrt{2}$ for the helicity basis *σ*_±_. Black areas in the quarter-disks then indicate that the modes have an almost pure degree of the corresponding polarization state. Although not enforced by our optimization approach, all modes associated with the highest eigenvalues can be characterized in terms of (M1) radial polarization, (M2) circular polarization, and (M3) azimuthal polarization. All circular modes are twofold degenerate (only one of them is shown), and one could alternatively perform a superposition to obtain modes with horizontal and vertical linear polarization. In the figure, we additionally show results for a (G) Gaussian incoming beam with circular polarization and will comment on its properties later. Note that the radial and azimuthal unit vectors form a basis, but the vectors are not orthogonal to *σ*_±_. For this reason, there is an approximately fifty percent *σ*_+_ contribution to the radial (M1) and azimuthal (M3) modes. One observes that modes M1 and M3 have zero intensity at *ρ* = 0, whereas the circular modes M2 and G exhibit a rather weak intensity variation along *ρ*.

Figure 3(b) and (b*) shows the intensities of the focused fields ***E***_inc_ in the plane of the scatterer that is located in the center of the panels. The radial mode M1 has a maximum intensity at the particle position ***R***_0_, where the electric field points in the *z*-direction, contrary to the azimuthal mode that has zero electric field intensity there. The intensity maxima of M2 and G are located at ***R***_0_, and the electric field lies in the *xy*-plane and has a circular polarization. In the panels, we also report the relative importance of the eigenvalues *D* = 100 Λ/Λ_max_, with a factor of *D* = 100 corresponding to the largest eigenvalue.

The dependence of the eigenvalues (that equals the quantum Fisher information) on the numerical aperture is shown in Figure 3(c) and (c*). For NA values below say 1.3, the circular mode M2 performs best; however, the Gaussian mode with our ad-hoc choice of a unit variance performs almost equally well. This is remarkable. We have developed a framework for optimizing the quantum Fisher information, enabling the computation of fields that maximize localization precision, and have not imposed any constraints on the shape of the incoming fields ***ϵ***_inc_. Also their polarization properties have been left arbitrary. Nevertheless, the diagonalization approach comes up with four “boring” modes to be classified in terms of radial, circular, and azimuthal polarization. And for NA ≤ 1.3, the performance is even comparable to that of a simple Gaussian beam. Alas, we had hoped for better.

Things change for larger NA values, in particular for the scatterer on top of the substrate. Here, the radial mode M1 has the largest eigenvalue, which further increases for NA values that are large enough to support incoming waves with propagation angles above the cutoff value for total internal reflection (TIR). These wave components generate evanescent fields above the glass–water interface, which induce a strong dipole moment of the scatterer in *z*-direction and correspondingly an efficient scattering into the substrate. Indeed, when comparing panels (c) and (c*) for the scatterer on top and above the interface, we observe that for the large gap distance of 1 μm evanescent waves play no role and the eigenvalues remain bound to those NA values where TIR waves can be excited. It is thus the appearance of evanescent waves that boosts the localization precision. We note that evanescent waves not only play a role for radial polarization but also for all other modes, see Figure 3(c), although the localization enhancement is by far the strongest for the M1 mode.

In Appendix A, we show that the optimized fields also perform well for particles located in the vicinity of the spot for which they have been optimized. We also demonstrate that the precise value of the scaling parameter *γ* is not overly important.

**Figure 4:**
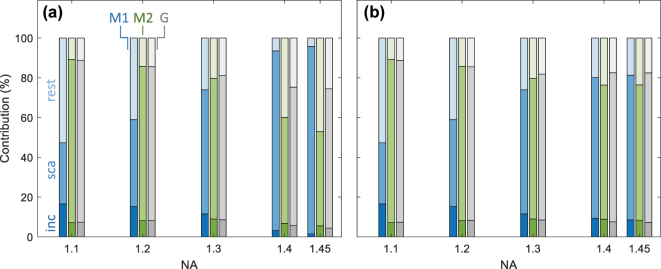
Incoming, scattered, and remaining contributions of quantum Fisher information for different modes and NA values, see Eq. (19), and for (M1) radial, (M2) circular, and (G) Gaussian mode. We show results for the scatterer located (a) on top and (b) 1 μm away from the interface. For details, see text.

Figure 4 provides a deeper insight into the contributions to the quantum Fisher information. The transition matrix in Eq. (12) can be conveniently decomposed into two contributions

(18) $$T\left(\theta \right)=T_{\text{sca}}\left(\theta \right)\cdot T_{\text{inc}}\left(\theta \right),$$

where ***T***_inc_(***θ***) accounts for the transition from the incoming fields to the scatterer, and ***T***_sca_(***θ***) for the transition from the scatterer to the backfocal plane. The terms entering Eq. (15) can then be decomposed into the contributions

(19) $$\begin{array}{ll} F_{\theta_{i}} & =4{\left[T_{\text{sca}}\cdot \left(\partial_{\theta_{i}}T_{\text{inc}}\right)\right]}^{†}\cdot \left[T_{\text{sca}}\cdot \left(\partial_{\theta_{i}}T_{\text{inc}}\right)\right]\\ & \;+4{\left[\left(\partial_{\theta_{i}}T_{\text{sca}}\right)\cdot T_{\text{inc}}\right]}^{†}\cdot \left[\left(\partial_{\theta_{i}}T_{\text{sca}}\right)\cdot T_{\text{inc}}\right]\\ & \;+4{\left[\left(\partial_{\theta_{i}}T_{\text{sca}}\right)\cdot T_{\text{inc}}\right]}^{†}\cdot \left[T_{\text{sca}}\cdot \left(\partial_{\theta_{i}}T_{\text{inc}}\right)\right]\\ & \;+4{\left[T_{\text{sca}}\cdot \left(\partial_{\theta_{i}}T_{\text{inc}}\right)\right]}^{†}\cdot \left[\left(\partial_{\theta_{i}}T_{\text{sca}}\right)\cdot T_{\text{inc}}\right]. \end{array}$$

The expression in the first line accounts for the change of the incoming fields upon variation of *θ*_*i*_ while keeping the scattering properties fixed (incoming contribution). This contribution is expected to be large for excitation fields with high spatial gradients, where a slightly displaced particle is excited differently. The expression in the second line accounts for the modified scattering properties for fixed incoming fields (scattering contribution). Likewise, the expressions in the third and fourth lines account for mixed contributions (rest). The relative importance of the respective contributions is reported in Figure 4. Importantly, for the best modes (M2 for small NA values, M1 for larger NA values), the largest contribution is the scattering one. This shows that the high localization precision of these modes is *not* due to the fact that the scatterer is excited by a highly structured light field where the excitation properties change significantly when varying the particle position. Rather the scattering channel plays the important role, where a change of the particle position ***R***_0_ leads to far-field modifications that carry the information about ***R***_0_. For large NA values and the particle on top of the substrate, also the evanescent fields at ***R***_0_ are transformed into waves propagating toward the lens, which then carry additional information about ***R***_0_.

The above discussion explains why certain modes are better suited for particle localization than others: the optimized modes essentially maximize the field strength (and correspondingly the induced dipole moments) at the scatterer positions. Depending on the NA value of the focusing lens, the induced dipole moments are either located in the interface plane (M2) or perpendicular to it (M1). At the same time, the distribution of the incoming fields is surprisingly simple and could be approximated by even simpler Gaussian mode profiles with circular or radial polarization without significantly deteriorating the localization precision.

### 3.3 Optimization of Fisher information

So far we have computed the incoming fields that optimize the quantum Fisher information of Eq. (14). We should stress that our approach is quite general, and correspondingly the localization precision bounds obtained are generally valid for any type of coherent localization microscopy. However, it is not clear whether these bounds can indeed be reached in actual experiments and, if this is the case, which technique and localization protocol should be used. In this section, we make a first step in answering these questions and evaluate the localization performance of iscat. We start by comparing the quantum and iscat Cramér–Rao bounds, Eqs. (7) and (10), using the fields obtained from the optimization of the quantum Fisher information, and then continue to optimize directly the iscat Fisher information of Eq. (6). In our following discussion, we only present results for a scatterer located on top of the interface. As has been shown above, for smaller NA values, say below 1.3, the results for scatterers on top and away from the interface are very similar. In setups with larger NAs additionally evanescent waves can be excited, which are of importance only for scatterers on top of the interface.

**Figure 5:**
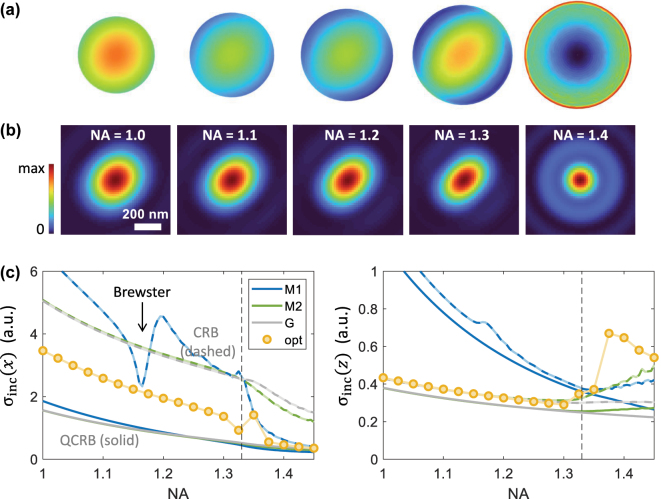
Optimization of the Fisher information for particle localization in iscat, see Eq. (6), and for a scatterer located on top of a glass–water interface. Electric field intensity in (a) backfocal plane and (b) plane of scatterer, and for the different NA values reported in the panels. (c) Localization precision along *x* (left panel) and *z* (right panel) as a function of the NA. The solid lines report the quantum Cramér–Rao bounds and the dashed lines the iscat Cramér–Rao bounds, as obtained from Eq. (7) for the optimized fields computed from the solutions of Eq. (14). The symbols show the bounds as obtained from the optimization of the Fisher information (*γ* = 0.2). For details, see text.

Figure 5 shows results for the iscat Cramér–Rao bounds, which are obtained by starting from the optimized solutions of the quantum Fisher information and maximizing the Fisher information in Eq. (5) using an iterative procedure. Similar results were also obtained when starting from a random guess for the incoming fields. Let us first concentrate on the dashed lines in panels (c), which report the NA dependence of $\sigma_{\text{inc}}^{\text{iSCAT}}$ for the localization precision along *x* (left) and *z* (right), using as incoming modes the solutions of the eigenvalue problem of Eq. (14) shown in Figure 3(a). With the exception of the dip around NA = 1.16, which is marked with an arrow and can be associated with waves close to the Brewster angle (a detailed discussion can be found in Ref. [28]), for NA values below 1.3 both the M2 mode with circular polarization and the Gaussian mode perform best. Things change for larger NA values, where the mode M1 with radial polarization achieves the best localization precision along *x*, however, at the expense of a lower precision along *z*. As has been discussed previously, this can be attributed to the excitation of evanescent waves at the glass–water interface.

When comparing the quantum (solid lines) and iscat (dashed lines) Cramér–Rao bounds, one observes for small NA values a ratio of about three for the localization precision along *x* (left) and a significantly smaller ratio for *z* (right). This finding is in agreement with those for plane-wave excitations [13], [40]. For NA values above 1.3, the ratio of the localization precision along *x* decreases, with values of 2.2 for NA = 1.4 and 1.7 for NA = 1.45. At the same time, the localization precision along *z* deteriorates.

We additionally performed optimizations for the iscat Fisher information given in Eq. (6). In the optimizations, we expanded the incoming fields in a basis obtained from the solutions of Eq. (14), keeping the 25 modes with highest eigenvalues, and optimized the expansion coefficients in an iterative procedure such that the Fisher information becomes maximized. We started the optimization either with the mode associated with the highest eigenvalue or with random expansion coefficients, obtaining similar results in both cases. Quite generally this doesn’t mean that our optimization approach provides us with the global maximum; however, it shows that it is not overly sensitive to the initial guess for the incoming fields.

The symbols in Figure 5 show the Cramér–Rao bounds obtained from these optimizations. One observes that $\sigma_{\text{inc}}^{\text{iSCAT}}\left(x\right)$ can be significantly reduced through optimization while keeping the precision along *z* almost unaltered, at least for NA values below 1.3. The optimized incoming field intensities are depicted in (a) the backfocal plane and (b) the plane of the scatterer. Closer inspection shows that for all NA values only a few basis modes with identical polarizations become mixed. As we don’t enforce any symmetry constraints in our optimizations, the modes originating from the iterative maximization procedure are slightly distorted and rotated. We have currently no clear explanation why these optimized modes perform better and cannot exclude that modes with an even better performance exist and will address these points in future work. A brief discussion of the scaling parameter *γ* in the context of the optimization results is given in Appendix A.

## 4 Summary and discussion

In summary, we have investigated the optimization of particle localization precision based on coherent scattering using structured light. Within the framework of the quantum Fisher information, we have derived a generalized eigenvalue problem whose solution provides us with the optimized excitation fields. In the context of the localization of small scatterers, these fields have relatively simple distributions and polarization properties and could even be replaced by more simple Gaussian fields. We have shown that the main physical mechanism of the optimization is the maximization of the number of scattered photons. In the context of interferometric scattering microscopy, we have discussed that the optimized fields outperform plane waves if the scatterer is prelocalized to within the focal spot, and the performance can be boosted through a further optimization based on the Fisher information.

Shaping the excitation light field, especially using radial polarization and high-NA focusing, can significantly improve localization precision in coherent microscopy. This has practical implications for enhancing localization precision in iscat experiments, particularly in label-free single-particle tracking, where nanometer-scale positional accuracy is essential. Importantly, our optimization suggests that, when phototoxicity is a problem, tracking should be done with focused light intensity, and not with wide-field illumination. Beyond these, the approach could also benefit biological imaging of nanoscale structures or dynamic processes where minimizing photon flux while maximizing spatial precision is critical.

For the generation of light beams featuring a ring-like intensity distribution and a radial or azimuthal polarization, standard liquid-crystal–based q-plates can be utilized, resulting in high-quality cylindrical vector beams. The use of spatial light modulators or bespoke metasurfaces for beam-shaping would allow for more complex beam shaping, not only structuring the polarization state but also the required intensity to match more closely the modes with optimized Fisher information.

The probably most appealing part of our work is the optimization of the quantum Fisher information through the solution of a generalized eigenvalue problem, following the seminal work of Ref. [24]. This approach not only provides us with a simple and versatile scheme to obtain general and definite answers, it also paves the way for a variety of other investigations using different optimization goals. In this context, the central ingredients are the matrices entering the eigenvalue problem, which encode the goals we are seeking for.

In this work, we have been seeking for the optimization of the incoming fields that allow for the best possible localization, subject to the constraint that the power of the incoming fields is kept constant. The power constraint could be modified, for instance such that the quantum Fisher information per detected photon is maximized, as is usually done in fluorescence microscopy, see also the introductory discussion. Here, the optimal incoming beam becomes the azimuthal mode, which is extremely sensitive to variations of the scatterer position located in close vicinity to the vortex center, despite the fact that only very few photons are scattered.

In a broader sense, all such types of optimization in optical microscopy call for precise questions of what should be optimized and how different setups should be compared. It often only takes a small shift in the optimization goals to get completely different answers. Although this is a somewhat trivial statement, we feel that it is sometimes not fully appreciated. If applied rigorously, the (quantum) Fisher information framework provides a powerful tool and can give definite answers in the context of optical microscopy.

## Figures and Tables

**Figure 6:**
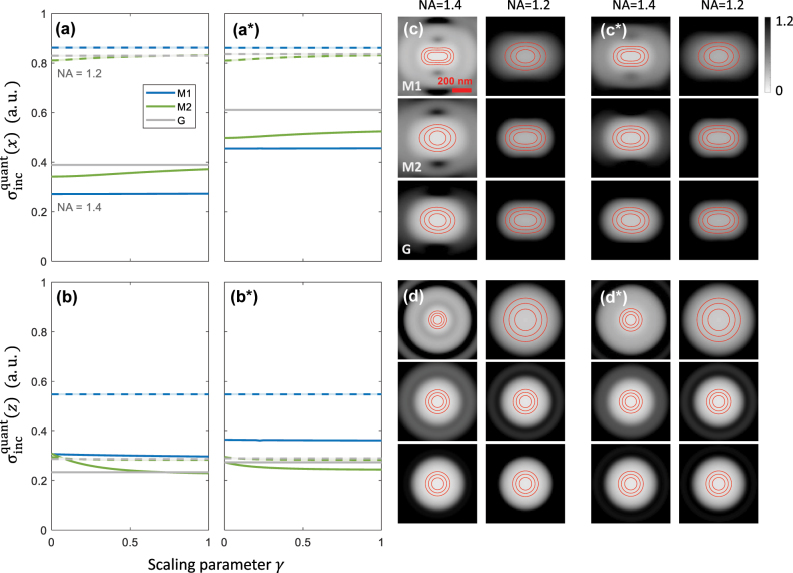
Dependence of quantum Cramér–Rao bound of Eq. (7) on the scaling parameter *γ* and the particle position ***R***_0_. Panels (a, b) report the scaling parameter dependence of the bound $\sigma_{\text{inc}}^{\text{quant}}$ (in arbitrary units) for a particle located on top of a glass–water interface, and for localization of (a) *X*_0_ and (b) *Z*_0_. We analyze the dependence for the different modes M1, M2, G. Panels (c, d) show maps of the bound $\sigma_{\text{inc}}^{\text{quant}}$ for particles located at different positions in the *xy*-plane and for localization of (a) *X*_0_ and (b) *Z*_0_. The red lines are isolines for *σ*_inc_ at values 10, 25, and 50 percent above its minimum value at the center. (a*–d*) Same as (a–d) but for particle located 1 μm above the interface.

**Figure 7:**
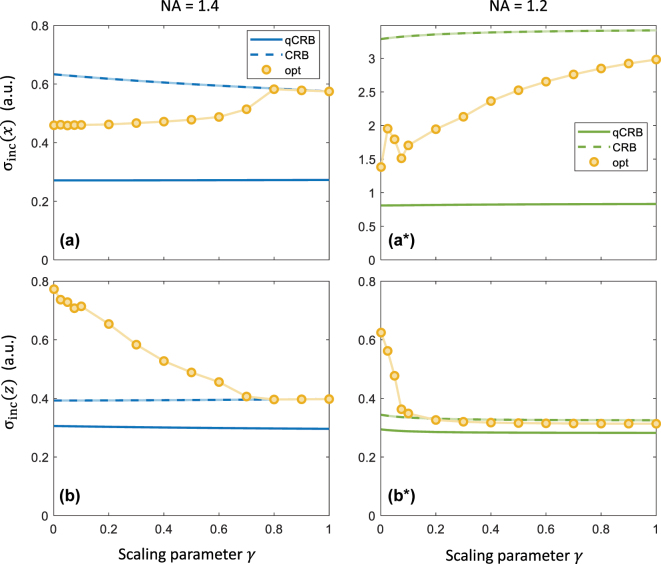
Dependence of quantum and iscat Cramér–Rao bound on the scaling parameter *γ*. Panels (a) and (b) show the dependence of the quantum (solid lines) and iscat (dashed lines) bounds for localization along *x* and *z*, respectively, and for NA = 1.4 and the mode M1 with radial polarization. The symbols report the bounds obtained from the optimization of the iscat Fisher information. (a*, b*) Same as (a, b) but for NA = 1.2.

## Appendix A: Robustness of simulation results

In this Appendix, we investigate the influence of our simulation results on the scaling parameter *γ* and show that the optimized fields also perform well for scatterer positions in the vicinity of the position for which the fields have been optimized.

Figure 6 shows the dependence of the quantum Cramér–Rao bound $\sigma_{\text{inc}}^{\text{quant}}$ of Eq. (10) on the scaling parameter *γ* and the particle position ***R***_0_. Panels (a–d) report results for a particle located on top of a glass–water interface, and panels (a*–d*) for a particle located 1 μm away from it. As for the scaling parameter, we find that it has only a minor influence on the localization precision bounds. In panels (c, d), we use the optimized fields for the scatterer located at ***R***_0_ = (0, 0, *Z*_0_) and investigate $\sigma_{\text{inc}}^{\text{quant}}$ for different particle positions ***R***_0_ = (*X*_0_, *Y*_0_, *Z*_0_). As can be seen from the maps, $\sigma_{\text{inc}}^{\text{quant}}$ doesn’t change significantly in the vicinity of ***R***_0_ = (0, 0, *Z*_0_). A similar behavior is found when varying the particle position in the *z* direction (not shown). This demonstrates that the optimized fields not only work at the exact position for which the fields have been designed but also in an extended neighborhood region.

In Figure 7, we investigate the dependence of $\sigma_{\text{inc}}^{\text{iSCAT}}$ obtained in iscat on the scaling parameter *γ*. For small *γ* values, the localization precision along *z* depends sensitively on *γ*. We recommend using *γ* values that give a good compromise between the localization precisions along *x* and *z*, in our simulations we set *γ* = 0.2 if not stated differently. As the localization precision along *z* is practically always better than that of *x*, we have refrained from investigating *γ* parameters above one.
